# Eco-friendly spectrofluorimetric and HPLC-fluorescence methods for simultaneous determination of melatonin and zolpidem in pharmaceuticals

**DOI:** 10.1038/s41598-025-18325-y

**Published:** 2025-09-18

**Authors:** Shrouk M. Abo Elkheir, Jenny Jeehan M. Nasr, Mohamed I. Walash, Abdallah M. Zeid

**Affiliations:** 1 Department of Pharmaceutical Analytical Chemistry, Faculty of Pharmacy, Mansoura University, Mansoura, 35516 Egypt; 2 Department of Pharmaceutical Analytical Chemistry, Faculty of Pharmacy, Mansoura National University, Gamasa, 7731168 Egypt

**Keywords:** Melatonin, Zolpidem tartrate, First derivative synchronous spectrofluorimetry, HPLC with fluorescence detection, Pharmaceutical analysis, Greenness assessment, Chemistry, Environmental sciences

## Abstract

Two eco-friendly, facile, rapid, and highly sensitive analytical methodologies were developed for the concurrent estimation of melatonin and zolpidem tartrate in raw materials and dosage forms. The first approach is a green first derivative synchronous spectrofluorimetric method (Method I), utilizing a wavelength interval (Δλ) of 60 nm and capturing derivative signals at 265.0 nm and 339.0 nm for melatonin and zolpidem, respectively. Method I exhibited excellent linearity over the ranges of 8.0–70.0 ng/mL for melatonin and 10.0–80.0 ng/mL for zolpidem, with detection limits of 1.62 ng/mL and 1.19 ng/mL. The second method (Method II) involves high-performance liquid chromatography coupled with fluorescence detection (HPLC-FD), employing a C18 column and a mobile phase of methanol:0.05% triethylamine (70:30, v/v, pH 5.5) at a flow rate of 1.0 mL/min. Fluorescence detection was performed at 383 nm following excitation at 243 nm. This method achieved linearity within 150.0–1500.0 ng/mL for melatonin and 50.0–700.0 ng/mL for zolpidem, with detection limits of 18.87 ng/mL and 8.86 ng/mL, respectively. Both methods were rigorously validated in accordance with ICH Q2(R1) guidelines, demonstrating high accuracy, precision, and robustness in analyzing commercial dosage forms. Greenness and sustainability were quantitatively assessed using the AGREE and eco-scale metrices, while method applicability and performance were evaluated using the Blue Applicability Grade Index (BAGI), affirming both techniques as efficient and environmentally sustainable tools for routine quality control.

**Supplementary Information:**

The online version contains supplementary material available at 10.1038/s41598-025-18325-y.

## Introduction

**Fig. 1:**
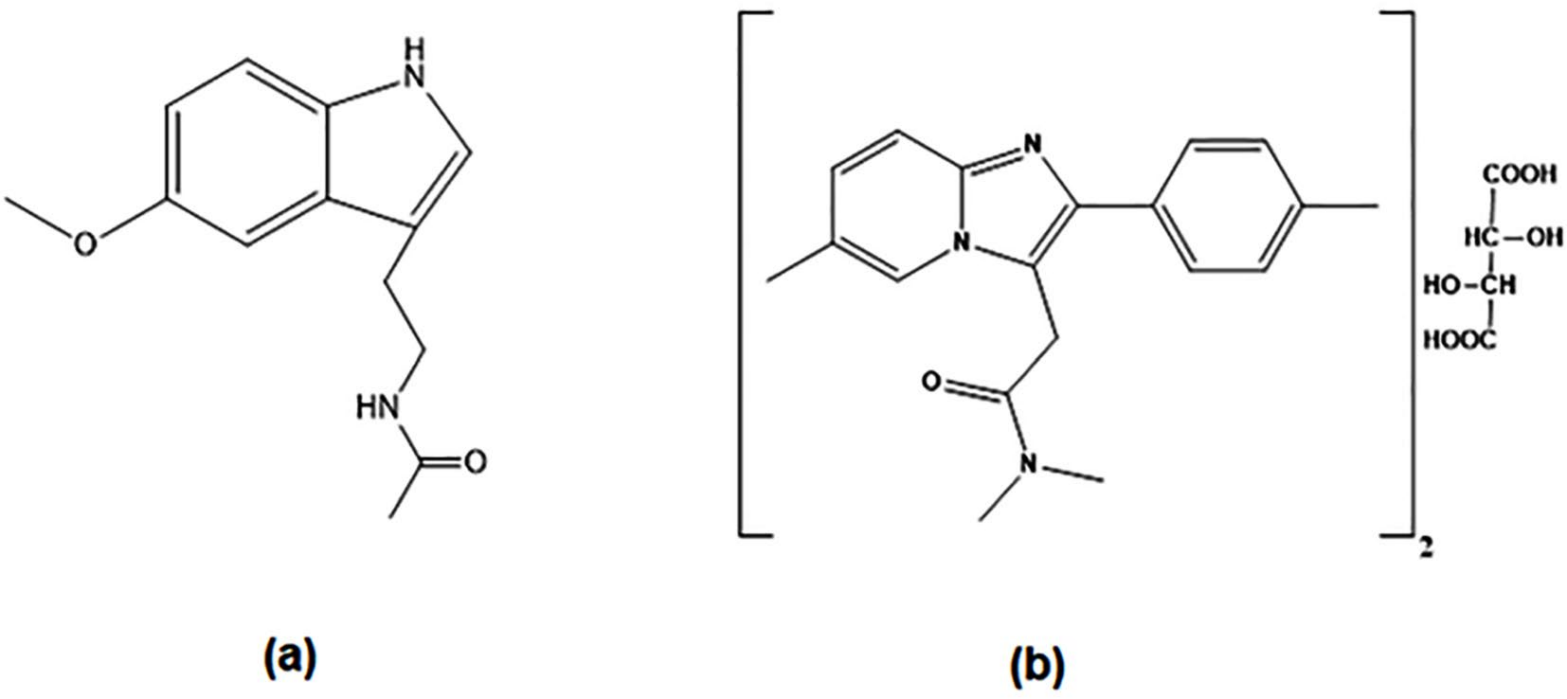
Chemical structure of (**a**) Melatonin and (**b**) Zolpidem Tartrate.

Melatonin (MLT) (Fig. 1a) is a hormone predominantly secreted by the pineal gland at night, playing a central role in regulating the circadian rhythm^1^. It is widely used in the treatment of sleep disorders, including jet lag^2^. Beyond its chronobiotic action, MLT exhibits antioxidant properties and pro-apoptotic effects in cancer cells, making it a valuable adjuvant in chemotherapy by enhancing efficacy and reducing toxicity^3^. Additional studies suggest its potential in managing various conditions such as irritable bowel syndrome, gastroesophageal reflux disease (GERD), Alzheimer’s disease, and inflammatory disorders^4–7^.

Both British Pharmacopeia (BP)^8^ and United State Pharmacopeia (USP)^9^ recommended HPLC method for the determination of MLT using phosphate buffer and acetonitrile as mobile phase. Other reported analytical techniques include spectrofluorimetry^10–13^, spectrophotometry^14–16^, HPLC^17–21^, capillary electrophoresis^22–24^, and electrochemical methods^25,26^.

Zolpidem tartrate (ZOL) (Fig. 1b), an imidazopyridine derivative with sedative properties, acts as a GABA_A receptor agonist and is commonly prescribed for short-term treatment of insomnia^27^. It offers benzodiazepine-like sedation with minimal anxiolytic or muscle relaxant effects. According to BP, ZOL can be determined by potentiometric titration using perchloric acid^8^, while the USP suggested a chromatographic method using methanol, acetonitrile, and buffer (23:18:59,v/v/v%) with UV detection at 254 nm^9^. Several analytical methods have also been published for the estimation of ZOL, including spectrophotometry^16,28–31^, HPLC^20,21,32–35^, capillary electrophoresis^36,37^, and electrochemical methods^38–41^.

MLT and ZOL are frequently co-administered in the management of insomnia, as MLT restores the natural sleep–wake cycle while ZOL promotes sleep induction. This combination has also shown therapeutic benefit in conditions such as cranial dystonia, particularly when conventional therapies are ineffective^42^.

Despite their pharmacological synergy and frequent co-prescription, analytical methods enabling their simultaneous quantification remain limited. Existing methods rely mainly on spectrophotometric^16^ or conventional HPLC techniques^20,21^, with no reported fluorescence-based approaches for their concurrent determination. This represents a significant analytical gap, considering the well-established advantages of fluorescence techniques in terms of sensitivity, selectivity, and environmental compatibility over UV-based methods^43,44^. They are also easy, precise and time saving techniques^45,46^.

In this study, we report two novel, green, and complementary fluorescence-based methods for the simultaneous determination of MLT and ZOL in pharmaceutical formulations: (1) a first derivative synchronous fluorescence spectrometric (FD-SFS) method, which enhances spectral resolution and selectivity by resolving overlapping fluorescence signals^47,48^; and (2) a high-performance liquid chromatography method with fluorescence detection (HPLC-FD), offering high sensitivity and efficient separation^49^. Both methods were validated in accordance with ICH Q2(R1) guidelines and demonstrated superior analytical performance compared to previously reported techniques (Table S1). Furthermore, the environmental sustainability and practical applicability of the proposed methods were evaluated using the AGREE (Analytical GREEnness) metric, analytical eco-scale and the Blue Applicability Grade Index (BAGI), confirming their suitability as high-throughput, green alternatives for routine pharmaceutical quality control.

## Experimental

### Instruments

Spectrofluorimetric measurements were performed using a Cary Eclipse fluorescence spectrophotometer equipped with a Xenon lamp (Agilent, Califormia, USA). The instrument was operated with a high voltage of 800 V, a smoothing factor of 20, and a slit width of 5 nm. First derivative spectra were generated using a filter size of 20 and a scan interval of 1 nm.

High-performance liquid chromatography (HPLC) analysis was conducted on an Agilent 1260 Infinity II system, which included a quaternary pump, an auto-sampler, and a fluorescence detector (Agilent, Califormia, USA).

A Consort pH meter (Model P-901, Belgium) was used for pH measurements, and a Vortex mixer (Model IVM-300p, Taiwan) was used for sample mixing during preparation.

### Materials and reagents

Melatonin (MLT) with a purity of 99.4% was obtained from Amoun Pharmaceutical Company, Egypt. Zolpidem tartrate (ZOL), with a purity of 99.3%, was supplied by Future Pharmaceutical Company, Egypt. The purities of both substances were confirmed by a previously reported comparison method^21^.

Dozova melatonin^®^ capsules (each containing 5 mg of melatonin), manufactured by ZETA PHARMA, Cairo, Egypt, were purchased from a local pharmacy for the study.

Solvents including methanol, ethanol, and acetonitrile were purchased from Fisher Scientific, UK. Surfactants and chemicals such as sodium dodecyl sulfate (SDS), Tween-80, cetrimide, boric acid, acetic acid (96%), sodium hydroxide, starch, talc, and lactose were sourced from El-Nasr Pharmaceutical Co. and PIOCHEM, Egypt.

Acetate buffer (0.2 M, pH 3.5–5.5) and borate buffer (0.2 M, pH 6.5–11) were freshly prepared as needed. Triethylamine (TEA) was purchased from ALPHA CHEMIKA, India, and orthophosphoric acid was obtained from Fisher Scientific, UK. A 0.2 M phosphoric acid solution was freshly prepared and used to adjust the pH to 5.5 in the HPLC method.

### Standards

Stock solutions of MLT and ZOL (100 µg/mL) were prepared in methanol. For the first-derivative synchronous spectrofluorimetric method (Method I), working solutions were prepared by diluting the stock solutions to 1 µg/mL with methanol. For the HPLC-fluorescence detection method (Method II), the stock solutions were diluted to 10 µg/mL with the mobile phase.

### HPLC conditions

Chromatographic separation was achieved using a HyperClone™ ODS C18 column (150 × 4.6 mm, 5 μm). The mobile phase consisted of methanol and 0.05% TEA (70:30, v/v), adjusted to pH 5.5. The flow rate was set at 1.0 mL/min. Detection was performed using a fluorescence detector set at an emission wavelength of 383 nm after excitation at 243 nm. The injection volume for all analyses was 10 µL.

### Procedures

#### Calibration curve construction

##### First-derivative synchronous spectrofluorimetry (Method Ⅰ)

To construct the calibration curves, a series of working standard solutions were prepared in methanol, covering concentration ranges of 8.0–70.0 ng/mL for melatonin (MLT) and 10.0–80.0 ng/mL for zolpidem (ZOL). Synchronous fluorescence spectra (SFS) were recorded at a wavelength offset (Δλ) of 60 nm. These spectra were then mathematically transformed into their first derivative (1D) form to enhance resolution. The 1D amplitude values were measured at 265.0 nm for MLT and 339.0 nm for ZOL. Calibration graphs were constructed by plotting these amplitudes against the corresponding drug concentrations, and the resulting regression equations were used for quantitative analysis.

##### HPLC with fluorescence detection method (Method ⅠⅠ)

For the HPLC-FD method, working standard solutions were prepared using the mobile phase as the diluent, within the ranges of 150–1500.0 ng/mL for MLT and 50.0–700.0 ng/mL for ZOL. Aliquots of 10 µL were injected into the HPLC system under the previously described chromatographic conditions. Calibration curves were obtained by plotting the peak areas against the corresponding concentrations, from which linear regression equations were derived.

#### Analysis of pharmaceutical formulations

Ten Dozova Melatonin^®^ capsules (each labeled to contain 5 mg of MLT) were opened, and the contents were accurately weighed. An amount equivalent to 10 mg of MLT was transferred to a 100 mL volumetric flask, dissolved in ~ 50 mL of methanol, and sonicated for 30 min to ensure complete extraction. The solution was then diluted to volume with methanol.

Laboratory-prepared ZOL tablets (10 mg/tablet) were formulated using zolpidem mixed with standard pharmaceutical excipients: 15 mg starch, 15 mg lactose, and 20 mg talc. A sample equivalent to one tablet was placed into a 100 mL volumetric flask, dissolved in methanol, sonicated for 30 min, and diluted to the mark. The mixture was filtered through Whatman No. 1 filter paper before analysis.

Additionally, laboratory-prepared co-formulated tablets containing MLT and ZOL in 3:5 and 3:10 ratios—based on the composition of the Indian formulation Zolsoma^®^—were similarly prepared by blending the two drugs with the same excipients in the specified proportions. Sample preparation followed the same protocol as described for the individual drugs. Analytical determination was then performed using both Method I (FD-SFS) and Method II (HPLC-FD) as described above.

## Results and discussion

The native fluorescence spectra of melatonin (MLT) and zolpidem (ZOL) exhibit emission maxima at 330.0 nm and 383.0 nm following excitation at 227.0 nm and 243.0 nm, respectively, in methanol (Figure S1). Exploiting this intrinsic property, two sensitive and facile fluorescence-based analytical techniques were developed for the simultaneous determination of MLT and ZOL. The first method utilizes synchronous fluorescence spectrofluorimetry (SFS) at a wavelength interval (Δλ) of 60 nm with methanol as the solvent. First derivative amplitudes were measured at 265.0 nm for MLT and 339.0 nm for ZOL, allowing effective resolution of their overlapping spectra. The second method involves chromatographic separation followed by fluorescence detection (HPLC-FD). Both approaches were systematically optimized and validated for the analysis of MLT and ZOL in pharmaceutical dosage forms. Additionally, their environmental sustainability and operational practicality were evaluated using AGREE, analytical eco scale and BAGI assessment tools.

### Optimisation of experimental parameters

#### Method I: first derivative synchronous spectrofluorimetry (FD-SFS)

**Fig. 2:**
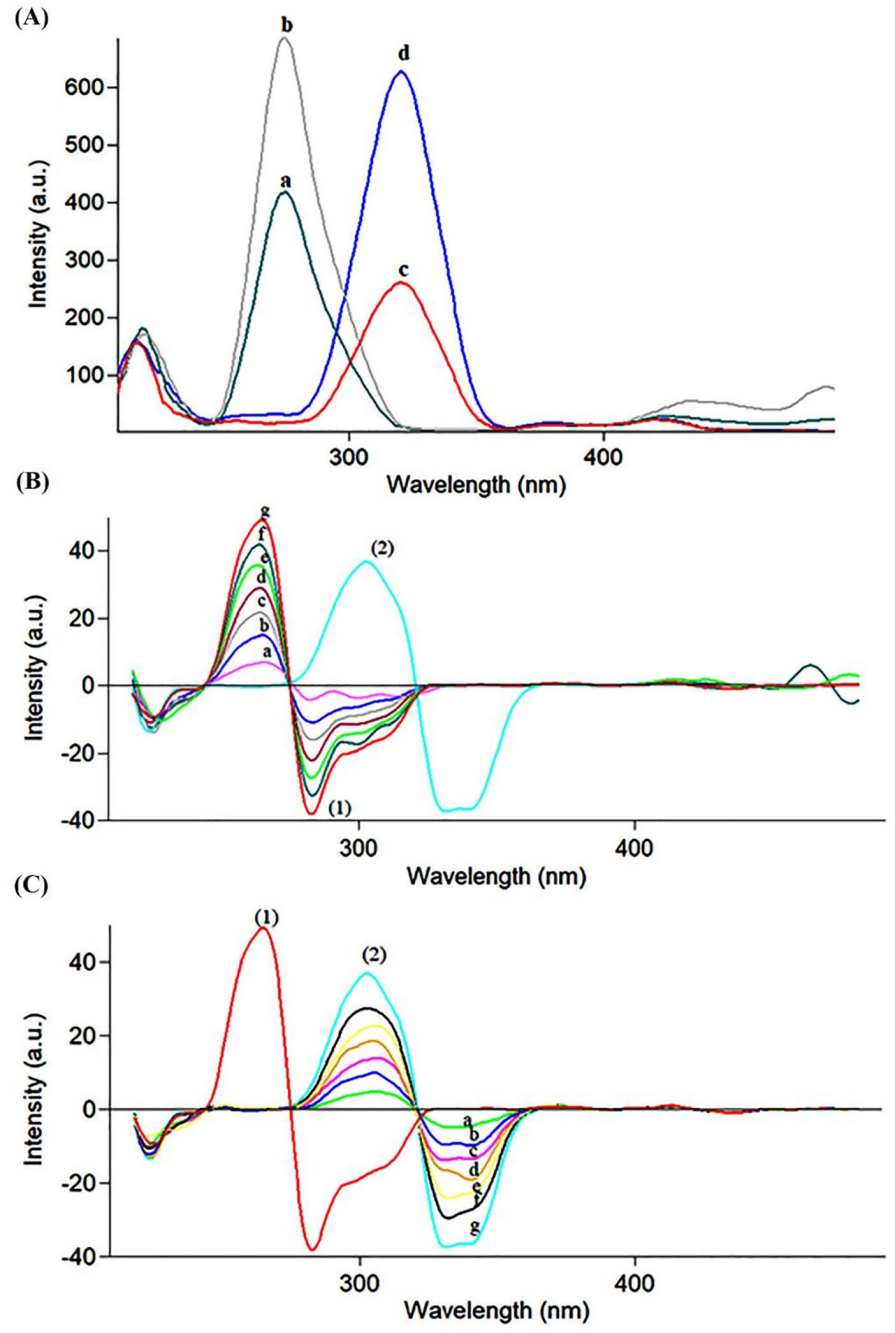
(**A**) Zero-order synchronous fluorescence spectra showing spectral overlap between MLT (a: 30.0, b: 50.0 ng·mL⁻¹) and ZOL (c: 20.0, d: 50.0 ng·mL⁻¹) at Δλ = 60 nm. (**B**) FD-SFS of (1) MLT at increasing concentrations (a–g: 8.0, 20.0, 30.0, 40.0, 50.0, 60.0, and 70.0 ng·mL⁻¹) and (2) ZOL (80.0 ng·mL⁻¹) measured at 265.0 nm. (**C**) FD-SFS of (1) MLT (70.0 ng·mL⁻¹) and (2) ZOL at increasing concentrations (a–g: 10.0, 20.0, 30.0, 40.0, 50.0, 60.0, and 80.0 ng·mL⁻¹) measured at 339 nm.

As depicted in Figure S1, the native emission spectra of MLT and ZOL exhibit significant overlap, impeding their direct simultaneous quantification. Figure 2A presents the synchronous fluorescence spectra of MLT and ZOL recorded at a Δλ of 60 nm. To enhance spectral resolution and enable selective quantification, first derivative processing of the synchronous spectra (FD-SFS) was applied (Fig. 2B, C). The critical experimental parameters affecting fluorescence intensity were systematically investigated and optimized as follows:

##### Selection of optimum Δλ

Synchronous spectra were recorded across a range of Δλ values (20–120 nm). Optimal sensitivity and spectral resolution were achieved at Δλ = 60 nm (Figure S2), which was selected for all subsequent measurements.

##### Effect of pH

The influence of pH on fluorescence intensity was assessed over the ranges of pH 3.5–5.5 using 0.2 M acetate buffer and pH 6.5–11 using 0.2 M borate buffer. Methanol alone produced significantly higher fluorescence intensities for both analytes compared to buffered solutions; thus, all further experiments were conducted in methanol without buffer, as illustrated in Figure S3.

##### Effect of surfactants

The effect of various surfactants—including sodium dodecyl sulfate (SDS), cetrimide, and Tween 80—was examined using water as the diluent. SDS and cetrimide slightly enhanced the fluorescence of ZOL but markedly quenched that of MLT. Tween 80 had no appreciable effect. Given these outcomes, no surfactant was used in the final method (Figure S3).

##### Effect of diluting solvents

Various solvents such as acetonitrile, methanol, ethanol and water were evaluated. Figure S3 shows that methanol was found to be the optimal solvent for ZOL. Although, acetonitrile yielding similar results, methanol was selected for further studies due to its cost-effectiveness and availability in addition to environmental reasons, making it the preferred solvent throughout the study.

#### Method ⅠⅠ: HPLC with fluorescence detection (HPLC-FD)

A reversed-phase HPLC-FD method was developed for the rapid, sensitive, and accurate quantification of melatonin (MLT) and zolpidem tartrate (ZOL) in pharmaceutical formulations. The chromatographic conditions were optimized to achieve efficient separation with sharp, symmetrical peaks and minimal tailing within a short run time.

Two types of columns were evaluated:

1. HyperClone™ MOS C8 (150 × 4.6 mm, 5 μm).
2. HyperClone™ ODS C18 (150 × 4.6 mm, 5 μm).

The C18 column was selected due to its superior performance in terms of peak shape and resolution.

Several chromatographic parameters were optimized, including mobile phase pH, the type and ratio of the organic modifier, the concentration of triethylamine (TEA), and the flow rate. The results of these evaluations are summarized in Table 1.

**Table 1:** Optimization of the chromatographic conditions for the determination of MLT and ZOL by HPLC-FD method.

Factor	Number of theoretical plates (NTP)		Tailing factor (T_f_)		Selectivity factor (ɑ)	Resolution (Rs)
	MLT	ZOL	MLT	ZOL		
pH of mobile phase						
3	2043	789	1.18	2.43	1.65	3.98
4	2035	1334	1.18	1.85	1.48	3.79
4.5	2109	2088	1.17	1.66	1.34	3.48
5	2070	2620	1.27	1.33	1.3	3.19
5.5	1962	2638	1.16	1.25	1.23	2.48
6	1969	2656	1.16	1.21	1.2	2.4
Type of organic modifier						
Acetonitrile	2030	2568	1.26	1.25	1.23	2.46
Methanol	2160	3491	1.18	1.21	1.6	6.1
Ethanol	1608	1810	1.32	1.23	1.16	1.5
Methanol: 0.05% TEA ratio						
60:40	1636	2767	0.76	1.27	2.45	10.04
65:35	2604	4098	1.39	1.34	1.86	8.77
70:30	1886	2748	0.99	1.3	1.55	5.15
75:25	2256	3474	2.34	1.32	1.39	4.32
80:20	1687	2539	1.32	1.34	1.22	2.22
Concentration of TEA						
0.05%	2014	2556	1.29	1.27	1.24	2.49
0.1%	2015	2507	1.1	1.26	1.23	2.47
0.2%	2030	2568	1.26	1.25	1.23	2.46
0.3%	2015	2587	1.21	1.28	1.23	2.4

Among the tested organic solvents (acetonitrile, methanol, and ethanol), methanol yielded the highest number of theoretical plates, best resolution, and lowest tailing factor, and was thus selected as the organic component of the mobile phase.

Initially, phosphate buffers at various pH levels were tested but resulted in poor peak shapes and unacceptable tailing for ZOL. To address this issue, TEA was added to the mobile phase. Different concentrations of TEA (0.05–0.3%) were investigated, and it was found that increasing TEA concentration had negligible effects on peak shape or resolution. Therefore, the lowest tested concentration (0.05%) was selected.

Various mobile phase ratios were also examined. A composition of methanol and 0.05% TEA (70:30, v/v) provided optimal separation with good resolution and minimal tailing in a reasonable analysis time.

The influence of pH was studied over the range of 3.0 to 6.0. Low pH values resulted in distorted peaks and higher tailing factors, whereas higher pH values improved peak symmetry. Consequently, pH 5.5 was chosen as optimal.

Flow rates ranging from 0.8 to 1.2 mL/min were evaluated. Since changes in flow rate had little impact on resolution or peak symmetry, a flow rate of 1.0 mL/min was selected for the best overall performance.

### Methods validation

The proposed methods were validated in accordance with the guidelines outlined by the International Council for Harmonisation (ICH) Q2(R1)^50^.

#### Linearity and range

Excellent linear relationships were observed for both methods. In Method I (FD-SFS), the fluorescence signal (first derivative amplitudes) showed strong linearity over the concentration ranges of 8.0–70.0 ng/mL for MLT and 10.0–80.0 ng/mL for ZOL. In Method II (HPLC-FD), linearity was established over the ranges of 150–1500 ng/mL for MLT and 50.0–700.0 ng/mL for ZOL. Correlation coefficients (r) were 0.9997 and 0.9999 for MLT in Method I and Method II, respectively, and 0.9999 for ZOL in both methods—indicating excellent linearity.

#### Limit of detection (LOD) and limit of quantitation (LOQ)

LOD and LOQ were calculated based on the standard deviation of the intercept (Sa) and the slope (b) using the equations^48^:

$${\text{LOD }} = {\text{ 3}}.{\text{3 }} \times {\text{ }}\left( {{\text{Sa}}/{\text{b}}} \right){\text{ and LOQ }} = {\text{ 1}}0{\text{ }} \times {\text{ }}\left( {{\text{Sa}}/{\text{b}}} \right)$$

- Method I (FD-SFS): LOD/LOQ were 1.62/4.90 ng/mL for MLT and 1.19/3.61 ng/mL for ZOL.
- Method II (HPLC-FD): LOD/LOQ were 18.78/57.17 ng/mL for MLT and 8.86/26.86 ng/mL for ZOL (Table S2).

These values confirm the high sensitivity of both methods, particularly the fluorescence-based technique.

#### Accuracy and precision

Accuracy was assessed by comparing the results of the proposed methods with a previously reported HPLC method using UV detection at 235 nm^21^. Statistical evaluation using Student’s *t*-test and *F*-test^51^ revealed no significant difference between methods, confirming the reliability of the proposed approaches (Table 2).

**Table 2:** Determination of the studied drugs in their raw materials using the proposed methods.

Parameters	MLT			ZOL		
	FD-SFS	HPLC-FD	Comparison method^19^	FD-SFS	HPLC-FD	Comparison method^19^
Percentage found^a^	100.81	100.04	100.68	98.83	100.87	100.72
	98.83	100.9	99.34	101.59	100.14	99.3
	99.35	98.75	100.19	98.22	101	100.23
	101.75	100.33		100.84	100.38	
	100.35	100.75		99.40	99.64	
	98.45	98.9		100.95	98.6	
	100.59	100.37		99.65	100.66	
Mean ± S.D.	100.02 ± 1.18	100.01 ± 0.86	100.07 ± 0.68	99.93 ± 1.23	100.18 ± 0.84	100.08 ± 0.72
t^b^	0.08 (2.31)*	0.12 (2.31)*		0.24 (2.31)*	0.19 (2.31)*	
F^b^	3.01 (19.33)*	1.6 (19.33)*		2.92 (19.33)*	1.36 (19.33)*	

^a^Average of 3 replicate determinations.

^b^The values between parentheses are the tabulated values of *t* and *F* at *P* = 0.05^51^.

Precision was evaluated at three concentration levels for each drug, both within a single day (intra-day) and across three consecutive days (inter-day). The relative standard deviation (RSD) values were consistently low, indicating excellent repeatability and intermediate precision (Table S3).

#### Selectivity

The proposed methods demonstrated high selectivity, as MLT and ZOL were accurately quantified in synthetic mixtures, laboratory-prepared co-formulated tablets, and commercial formulations without interference (Table S4). For FD-SFS, well-resolved derivative peaks were observed at 265.0 nm (MLT) and 339.0 nm (ZOL), as illustrated in Fig. 2B, C.

#### Robustness

Robustness was evaluated by introducing minor variations in key chromatographic parameters, including pH (5.5 ± 0.2), methanol composition (70 ± 2%), and triethylamine (TEA) concentration (0.05 ± 0.01%). These changes did not significantly impact retention times or peak resolution, confirming the robustness of Method II.

#### System suitability

System suitability was assessed based on USP and ICH criteria^9,50^. Parameters such as resolution, selectivity factor, tailing factor, number of theoretical plates, and retention times met the required specifications (Table S5), demonstrating that the system is suitable for routine quality control.

### Applications to pharmaceutical formulations

**Fig. 3:**
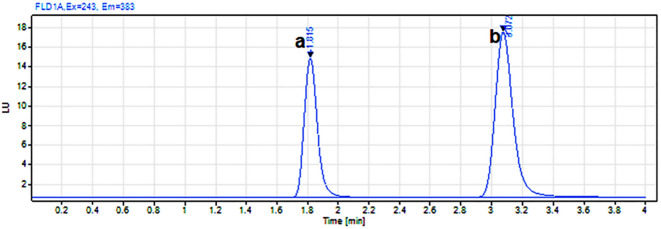
Representative HPLC-FD chromatogram of a mixture containing (**a**) MLT (900 ng·mL⁻¹) and (**b**) ZOL (200 ng·mL⁻¹).

The validated methods were successfully applied to the simultaneous determination of MLT and ZOL in synthetic mixtures (Table S4, Figure S5, Fig. 3), single-drug dosage forms (Table S6), and laboratory-prepared co-formulated tablets (Table S7). High recovery rates and consistent results were obtained with no interference with tablet excipients. Comparisons with the previously reported HPLC method^21^ using *t*- and *F*-tests showed no statistically significant differences, confirming the accuracy and practical applicability of the proposed methods.

### Greenness evaluation

**Fig. 4:**
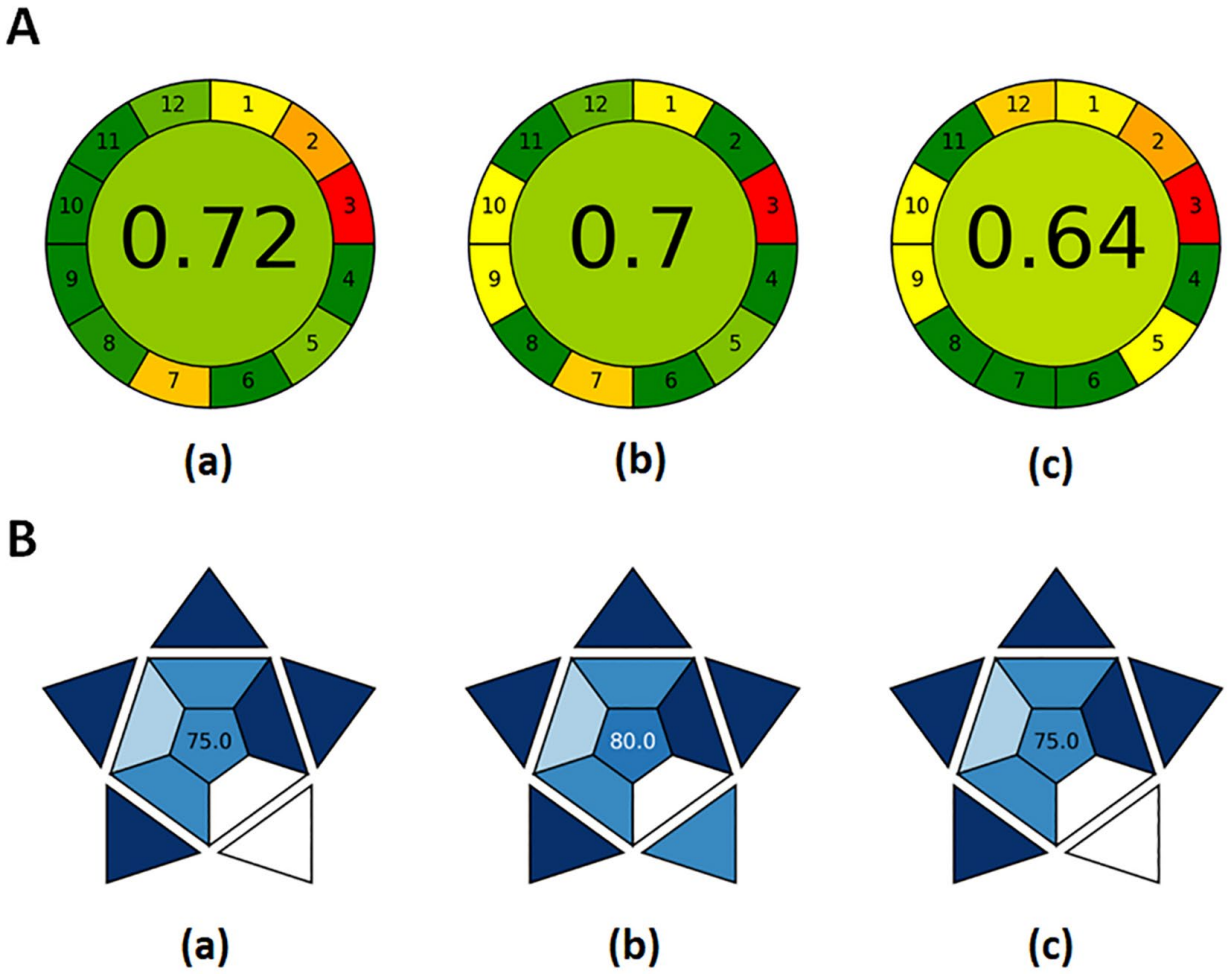
(**A**) AGREE pictograms of the proposed FD-SFS method (a), the proposed HPLC-FD method (b), and the published HPLC method (c). (**B**) BAGI pictograms of the proposed FD-SFS method (a), the proposed HPLC-FD method (b), and the published HPLC method (c).

The environmental impact of the developed methods was evaluated using the Analytical eco-scale and the Analytical GREEnness (AGREE) metric.which assesse compliance with the 12 principles of Green Analytical Chemistry (GAC). The eco-scale starts with a score of 100 from which the penalty points are subtracted^52^. For the analytical eco-scale the total score was 82 and 81 for FD-SFS and HPLC-FD, respectively (Table S8) .The AGREE pictogram displays 12 sectors and a core score ranging from 0 (poor) to 1 (ideal)^53–58^. As shown in Fig. 4, both developed methods received higher AGREE scores and exhibited more “green” sectors compared to the reference HPLC method, highlighting their superior eco-friendliness.

### Blueness evaluation

The practical applicability of the proposed methods was assessed using the Blue Applicability Grade Index (BAGI), a novel visual tool indicating method usability and reliability^59^. The blue-shaded pictogram provides a central score, where higher values indicate greater suitability for routine analysis as the method is considered good in applicability when it is lied in the range of 60–100 with excellent performance at score 100. As depicted in Fig. 4, the FD-SFS and HPLC-FD methods achieved BAGI scores of 75 and 80, respectively, reflecting their strong potential for real-world pharmaceutical applications.

## Conclusion

Two eco-friendly, sensitive, and rapid analytical methods—first derivative synchronous spectrofluorimetry (FD-SFS) and HPLC with fluorescence detection (HPLC-FD)—were developed and validated for the simultaneous quantification of melatonin and zolpidem in bulk and pharmaceutical forms. Both methods offer high precision, accuracy, and sensitivity, with minimal sample preparation and environmentally sustainable protocols. These methods were simple, cost effective and their superior greenness (AGREE) and practical applicability (BAGI) scores underscore their suitability for routine quality control, therapeutic drug monitoring, and forensic analysis—particularly valuable given zolpidem’s status as a controlled hypnotic agent.

## Supplementary Information

Below is the link to the electronic supplementary material.

Supplementary Material 1

## Data Availability

Data is provided within the manuscript or supplementary information files.

## References

[1] Arendt, J. & Skene, D. J. Melatonin as a chronobiotic. *Sleep Med. Rev.* **9**, 25–39 (2005). 15649736 10.1016/j.smrv.2004.05.002

[2] Csernus, V. & Mess, B. Biorhythms and pineal gland. *Neuroendocrinol. Lett.* **24**, 404–411 (2003). 15073565

[3] Lissoni, P. et al. Decreased toxicity and increased efficacy of cancer chemotherapy using the pineal hormone melatonin in metastatic solid tumour patients with poor clinical status. *Eur. J. Cancer*. **35**, 1688–1692 (1999). 10674014 10.1016/s0959-8049(99)00159-8

[4] Song, G. H., Leng, P. H., Gwee, K. A., Moochhala, S. M. & Ho, K. Y. Melatonin improves abdominal pain in irritable bowel syndrome patients who have sleep disturbances: a randomised, double blind, placebo controlled study. *Gut* **54**, 1402–1407 (2005). 15914575 10.1136/gut.2004.062034 PMC1774717

[5] Pereira, R. S. Regression of gastroesophageal reflux disease symptoms using dietary supplementation with melatonin, vitamins and aminoacids: comparison with Omeprazole. *J. Pineal Res.* **41**, 195–200 (2006). 16948779 10.1111/j.1600-079X.2006.00359.x

[6] Roy, J. et al. Role of melatonin in alzheimer’s disease: from preclinical studies to novel melatonin-based therapies. *Front. Neuroendocr.* **65**, 100986 (2022). 10.1016/j.yfrne.2022.100986 35167824

[7] Cho, J. H., Bhutani, S., Kim, C. H. & Irwin, M. R. Anti-inflammatory effects of melatonin: A systematic review and meta-analysis of clinical trials. *Brain. Behav. Immun.* **93**, 245–253 (2021). 33581247 10.1016/j.bbi.2021.01.034 PMC7979486

[8] *The British Pharmacopoeia* (The Stationary Office, 2013).

[9] U.S.P. Convention. *USP 33 NF 28: United States Pharmacopeia [and] National Formulary* (United States Pharmacopeial Convention, 2010).

[10] Pucci, V., Ferranti, A., Mandrioli, R. & Raggi, M. A. Determination of melatonin in commercial preparations by micellar electrokinetic chromatography and spectrofluorimetry. *Anal. Chim. Acta*. **488**, 97–105 (2003).

[11] Sorouraddin, M. H., Rashidi, M. R., Ghorbani-Kalhor, E. & Asadpour-Zeynali, K. Simultaneous spectrofluorimetric and spectrophotometric determination of melatonin and pyridoxine in pharmaceutical preparations by multivariate calibration methods. *Il Farmaco*. **60**, 451–458 (2005). 15885688 10.1016/j.farmac.2005.03.009

[12] Darwish, H. W., Attia, M. I. & Zlotos, D. P. New spectrofluorimetric methods for determination of melatonin in the presence of N-{2-[1-({3-[2-(acetylamino) ethyl]-5-methoxy-1H-indol-2-yl} methyl)-5-methoxy-1H-indol-3-yl]-ethyl} acetamide: a contaminant in commercial melatonin preparations. *Chem. Cent. J.* **6**, 1–11 (2012). 22551394 10.1186/1752-153X-6-36 PMC3778849

[13] Kumar, H. & Obrai, S. Ratiometric fluorescent sensing of melatonin based on inner filter effect and smartphone established detection. *Spectrochim. Acta Part A Mol. Biomol. Spectrosc.* **304**, 123309 (2024). 10.1016/j.saa.2023.123309 37716042

[14] Amin, A. S., Zaky, M. & El-Beshbeshy, A. M. Colorimetric Estimation of melatonin in pharmaceutical formulations. *Microchim. Acta*. **135**, 81–85 (2000).

[15] Uslu, B., Özkan, S. A. & Aboul-Enein, H. Y. Spectrophotometric determination of melatonin and pyridoxine HCL in binary mixture using first derivative of the ratio spectra method. *Anal. Lett.* **35**, 2305–2317 (2002).

[16] Venkatachalam, T. & Lalitha, K. Spectrophotometric methods for simultaneous estimation of melatonin and zolpidem from the combined tablet dosage form. *Pharmacophore* **5** (2014).

[17] Lin, J., Zhang, C., Gao, Y., Zhao, X. & Li, X. A validated HPLC method for determining melatonin in capsule dosage form. *Spatula DD*. **2**, 147–151 (2012).

[18] Vitale, A. A., Ferrari, C. C., Aldana, H. & Affanni, J. M. Highly sensitive method for the determination of melatonin by normal-phase high-performance liquid chromatography with fluorometric detection. *J. Chromatogr. B Biomed. Sci. Appl.* **681**, 381–384 (1996). 10.1016/0378-4347(96)00051-5 8811450

[19] Azİzoğlu, G. A., Azİzoğlu, E., Tanriverdİ, S. T. & Özgen, Ö. A validated HPLC method for simultaneous Estimation of melatonin and octyl methoxycinnamate in combined pharmaceutical applications. *Marmara Pharm. J.* **21**, 921–930 (2017).

[20] Akula, G., Talari, Y., Phanindra, S. & Akula, G. Method development and validation for simultaneous Estimation of melatonin and Zolpidem tartrate by using RP-HPLC. *Sch. Acad. J. Pharm.* **4**, 240–244 (2015).

[21] Sattar, A. & Suneetha, A. A validated RP-HPLC method for the determination of melatonin and Zolpidem tartarate in bulk and pharmaceutical dosage forms. *Int. Res. J. Pharm.* **9**, 90–96 (2018).

[22] Chen, G., Ding, X., Cao, Z. & Ye, J. Determination of melatonin and pyridoxine in pharmaceutical preparations for health-caring purposes by capillary electrophoresis with electrochemical detection. *Anal. Chim. Acta*. **408**, 249–256 (2000).

[23] Poboży, E., Michalski, A., Sotowska-Brochocka, J. & Trojanowicz, M. Determination of melatonin and its precursors and metabolites using capillary electrophoresis with UV and fluorometric detection. *J. Sep. Sci.* **28**, 2165–2172 (2005). 16318213 10.1002/jssc.200500095

[24] Hevia, D. et al. Development and validation of new methods for the determination of melatonin and its oxidative metabolites by high performance liquid chromatography and capillary electrophoresis, using multivariate optimization. *J. Chromatogr. A*. **1217**, 1368–1374 (2010). 20079907 10.1016/j.chroma.2009.12.070

[25] Gomez, F. J. V., Martín, A., Silva, M. F. & Escarpa, A. Screen-printed electrodes modified with carbon nanotubes or graphene for simultaneous determination of melatonin and serotonin. *Microchim. Acta*. **182**, 1925–1931 (2015).

[26] Apetrei, I. M. & Apetrei, C. Voltammetric determination of melatonin using a graphene-based sensor in pharmaceutical products. *Int. J. Nanomed.* **11**, 1859 (2016). 10.2147/IJN.S104941 PMC4859415 27194909

[27] Sweetman, S. C. *Martindale: The Complete Drug Reference* (Pharmaceutical Press, 2009).

[28] Chomwal, R., Kumar, A. & Goyal, A. Spectrophotometric methods for determination of Zolpidem tartrate in tablet formulation. *J. Pharm. Bioallied Sci.* **2**, 365 (2010). 21180474 10.4103/0975-7406.72142 PMC2996077

[29] Patil, K., Pore, Y. & Bhise, S. Spectrophotometric Estimation of Zolpidem in tablets. *J. Pharm. Sci. Res.* **2**, 1 (2010).

[30] Annapurna, M. M., Swathi, B., Chandra, M. S. & Tulasi, K. Derivative spectrophotometric methods for the determination of Zolpidem tartrate in tablets. *J. Appl. Pharm. Sci.* **2**, 096–099 (2012).

[31] Naidu, M., Venkatesh, D. & Chakravarthy, I. Spectrophotometric analysis of Zolpidem tartrate in pharmaceutical formulation. *Res. J. Pharm. Technol.* **4**, 917–919 (2011).

[32] Guinebault, P., Dubruc, C., Hermann, P. & Thénot, J. High-performance liquid chromatographic determination of zolpidem, a new sleep inducer, in biological fluids with fluorimetric detection. *J. Chromatogr. B Biomed. Sci. Appl.* **383**, 206–211 (1986). 10.1016/s0378-4347(00)83462-3 3818839

[33] Durol, A. L. B. & Greenblatt, D. J. Analysis of Zolpidem in human plasma by high-performance liquid chromatography with fluorescence detection: application to single-dose Pharmacokinetic studies. *J. Anal. Toxicol.* **21**, 388–392 (1997). 9288593 10.1093/jat/21.5.388

[34] Ring, P. R. & Bostick, J. M. Validation of a method for the determination of Zolpidem in human plasma using LC with fluorescence detection. *J. Pharm. Biomed. Anal.* **22**, 495–504 (2000). 10766367 10.1016/s0731-7085(99)00311-8

[35] Yaripour, S., Rashid, S. N., Alibakhshi, H. & Mohammadi, A. Development and validation of a stability-indicating reversed phase HPLC method for the quality control of Zolpidem in bulk and tablet dosage forms. *J. Anal. Chem.* **70**, 738–743 (2015).

[36] Hempel, G. & Blaschke, G. Direct determination of Zolpidem and its main metabolites in urine using capillary electrophoresis with laser-induced fluorescence detection. *J. Chromatogr. B Biomed. Sci. Appl.* **675**, 131–137 (1996). 10.1016/0378-4347(95)00342-8 8634754

[37] Al Azzam, K. M., Yit, L. K., Saad, B. & Shaibah, H. Development and validation of a stability-indicating capillary electrophoresis method for the determination of Zolpidem tartrate in tablet dosage form with positive confirmation using 2D-and 3D-DAD fingerprints. *Sci. Pharm.* **82**, 341–356 (2014). 24959406 10.3797/scipharm.1401-11 PMC4065127

[38] Radi, A. E., Bekhiet, G. & Wahdan, T. Electrochemical study of Zolpidem at glassy carbon electrode and its determination in a tablet dosage form by differential pulse voltammetry. *Chem. Pharm. Bull.* **52**, 1063–1065 (2004). 10.1248/cpb.52.1063 15340190

[39] Naeemy, A., Sedighi, E. & Mohammadi, A. Electrooxidation of Zolpidem and its voltammetric quantification in standard and pharmaceutical formulation using pencil graphite electrode. *J. Electrochem. Sci. Technol.* **7**, 68–75 (2016).

[40] Naghian, E. & Sohouli, E. A new electrochemical sensor for determination of Zolpidem by carbon paste electrode modified with sns@ SnO2NP. *Anal. Bioanal. Electrochem.* **12**, 458–467 (2020).

[41] Sohouli, E. et al. Sensitive sensor based on TiO2NPs nano-composite for the rapid analysis of zolpidem, a psychoactive drug with cancer-causing potential. *Mater. Today Commun.* **26**, 101945 (2021).

[42] Barad, N. H. & Mulroy, E. Successful treatment of cranial dystonia using a Zolpidem + Melatonin combination: A video case-series. *J. Neurol. Sci.* **460**, 122986 (2024). 38583390 10.1016/j.jns.2024.122986

[43] El-Din, M. S., Eid, M. & Zeid, A. M. Simultaneous determination of methocarbamol and aspirin by RP-HPLC using fluorescence detection with time programming: its application to pharmaceutical dosage form. *Luminescence* **28**, 332–338 (2013). 22715138 10.1002/bio.2386

[44] Abd El-Aziz, H. & Zeid, A. M. Derivatization-free conventional and synchronous spectrofluorimetric Estimation of Atenolol and amlodipine. *Spectrochim. Acta Part A Mol. Biomol. Spectrosc.* **305**, 123532 (2024). 10.1016/j.saa.2023.123532 37864972

[45] El Hamd, M. A., Mahdi, W. A., Alshehri, S., Alsehli, B. R. & Abu-Hassan, A. A. Streamlined turn-off fluorescence sensing of donepezil: enhancing evaluation in pharmaceutical formulations, quality control labs, and biological fluids at nano levels. *Spectrochim. Acta Part A Mol. Biomol. Spectrosc.* **327**, 125344 (2025). 10.1016/j.saa.2024.125344 39488081

[46] Abu-hassan, A. A. et al. Spectacular application of Isoindole‐Based fluorophore for mirabegron assay in tablets and biological fluids (Urine/Plasma). Assessment of method blueness and whiteness. *Luminescence* **40** (2), e70120 (2025). 39929498 10.1002/bio.70120

[47] Aboshabana, R., Zeid, A. M. & Ibrahim, F. A. Label-free green Estimation of Atenolol and ivabradine hydrochloride in pharmaceutical and biological matrices by synchronous spectrofluorimetry. *Spectrochim. Acta Part A Mol. Biomol. Spectrosc.* **295**, 122626 (2023). 10.1016/j.saa.2023.122626 36940537

[48] Abo Elkheir, S. M., Zeid, A. M., Nasr, J. J. M. & Walash, M. I. First derivative synchronous spectrofluorimetric analysis of Bisoprolol fumarate and ivabradine in pharmaceutical and biological matrices. Investigation of the method greenness. *Luminescence*. **37**, 1657–1665 (2022). 35834392 10.1002/bio.4337

[49] El-Din, M. S., Eid, M. & Zeid, A. M., simultaneous determination of methocarbamol and ibuprofen in their binary mixtures using Hplc method with fluorescence detection: application to combined tablets. *J. Liq. Chromatogr. Relat. Technol.* **36**, 852–866 (2013).

[50] *Q.R. ICH Harmonized Tripartite Guideline. Validation of Analytical Procedures: Text and Methodology, Current Step 4 Version, Parent Guidelines on Methodology Dated November 6 (1996)* (2005).

[51] Miller, J. & Miller, J. C. *Statistics and Chemometrics for Analytical Chemistry* (Pearson Education, 2018).

[52] Gałuszka, A., Migaszewski, Z. M., Konieczka, P. & Namieśnik, J. Analytical Eco-Scale for assessing the greenness of analytical procedures. *Trends Anal. Chem.* **37**, 61–72 (2012).

[53] Pena-Pereira, F., Wojnowski, W. & Tobiszewski, M. AGREE—Analytical greenness metric approach and software. *Anal. Chem.* **92**, 10076–10082 (2020). 32538619 10.1021/acs.analchem.0c01887 PMC7588019

[54] Zeid, A. M., El-Masry, A. A., El-Wasseef, D. R., Eid, M. & Shehata, I. A. Green microemulsion electrokinetic chromatographic method for simultaneous determination of azelastine and Budesonide. *Sustainable Chem. Pharm.* **29**, 100795 (2022).

[55] El Hamd, M. A. et al. Factorial design-aided derivatization-free fluorimetric ultrasensitive assay of Vonoprazan with application in uniformity of dosage units and plasma samples analysis: comprehensive and comparative greenness and whiteness assessment. *Microchem. J.* **205**, 111320 (2024).

[56] Abu-Hassan, A. A. Nano-level assay of attention-deficit/hyperactivity disorder medicament, Atomoxetine by molecular-size-based resonance Rayleigh scattering strategy. Employment in content uniformity, dosage form, and plasma analysis. *BMC Chem.* **17** (1), 175 (2023). 38057838 10.1186/s13065-023-01094-y PMC10702123

[57] Abu-hassan, A. A., Mahdi, W. A., Alshehri, S., Alsehli, B. R. & El Hamd, M. A. Innovative, eco-friendly fluorescence-quenching strategy for precise cyclopentolate evaluation in eye drops and aqueous humor-based enhanced ophthalmic care. *J. Photochem. Photobiol., A*. **462**, 116203 (2025).

[58] Abu-Hassan, A. A., Mahdi, W. A., Alshehri, S., Amin, M. M. & El Hamd, M. A. Facile and green chemistry-compatible fluorescence spectroscopic applications of acid red 87 used to evaluate eletriptan, antimigraine, in its pharmaceutical and biological samples. *Spectrochim. Acta Part A Mol. Biomol. Spectrosc.* **317**, 124400 (2024). 10.1016/j.saa.2024.124400 38710139

[59] Manousi, N., Wojnowski, W., Płotka-Wasylka, J. & Samanidou, V. Blue applicability grade index (BAGI) and software: a new tool for the evaluation of method practicality. *Green Chem.* **25**, 7598–7604 (2023).

