# Supplementary material for: Eco-friendly spectrofluorimetric and HPLC-fluorescence methods for simultaneous determination of melatonin and zolpidem in pharmaceuticals
Source: Sci Rep. 2025 Sep 18;15:32598. doi: 10.1038/s41598-025-18325-y (PMC12446487; doi:10.1038/s41598-025-18325-y)
Supplement: Supplementary file 1 — Supplementary Material 1 [file 41598_2025_18325_MOESM1_ESM.doc]

**Eco-Friendly Spectrofluorimetric and HPLC-Fluorescence Methods for Simultaneous Determination of Melatonin and Zolpidem in Pharmaceuticals**

**Shrouk M. Abo Elkheir^a*^, Jenny Jeehan M. Nasr^a,b^, Mohamed I. Walash^a^, and Abdallah M. Zeid^a^**

^a^Department of Pharmaceutical Analytical Chemistry, Faculty of Pharmacy, Mansoura University, Mansoura 35516, Egypt.

^b^Department of Pharmaceutical Analytical Chemistry, Faculty of Pharmacy, Mansoura National University, Gamasa 7731168, Egypt.

*Corresponding author: Shrouk M. Abo Elkheir, E-mail: shrouk@mans.edu.eg

Supplementary Material

| **Item** | **Page** |
| --- | --- |
| **Figure S1.** Excitation and emission spectra of MLT (20.0 ng·mL⁻¹) (a, a’) and ZOL (10.0 ng·mL⁻¹) (b, b’) in methanol. | S3 |
| **Figure S2.** Three-dimensional (3D) fluorescence spectra illustrating the effect of different Δλ values on (a) MLT and (b) ZOL. | S4 |
| **Figure S3.** Influence of (a) pH, (b) surfactants, and (c) solvents on the relative fluorescence intensity (RFI) of MLT and ZOL (10.0 ng·mL⁻¹ each). | S5 |
| **Figure S4.** First derivative synchronous fluorescence spectra of: (1) MLT (30.0 ng·mL⁻¹), (2) ZOL (50.0 ng·mL⁻¹), and (3) a mixture of MLT (30.0 ng·mL⁻¹) and ZOL (50.0 ng·mL⁻¹). | S6 |
| **Table S1**. Comparison of the analytical performance of the proposed method with previously reported methods | S7 |
| **Table S2.** Analytical performance characteristics of the proposed methods. | S8 |
| **Table S3.** Repeatability and intermediate precision of the proposed methods for MLT and ZOL. | S9 |
| **Table S4.** Determination of MLT and ZOL in synthetic mixtures using the proposed methods. | S10 |
| **Table S5.** System suitability parameters for the proposed HPLC method. | S11 |
| **Table S6.** Application of the proposed method to the determination of MLT and ZOL in their pharmaceutical formulations. | S12 |
| **Table S7.** Determination of MLT and ZOL in the prepared co-formulated tablet | S13 |
| **Table S8.** Analytical eco-scale of the proposed methods | S14 |
| **References** | S15 |


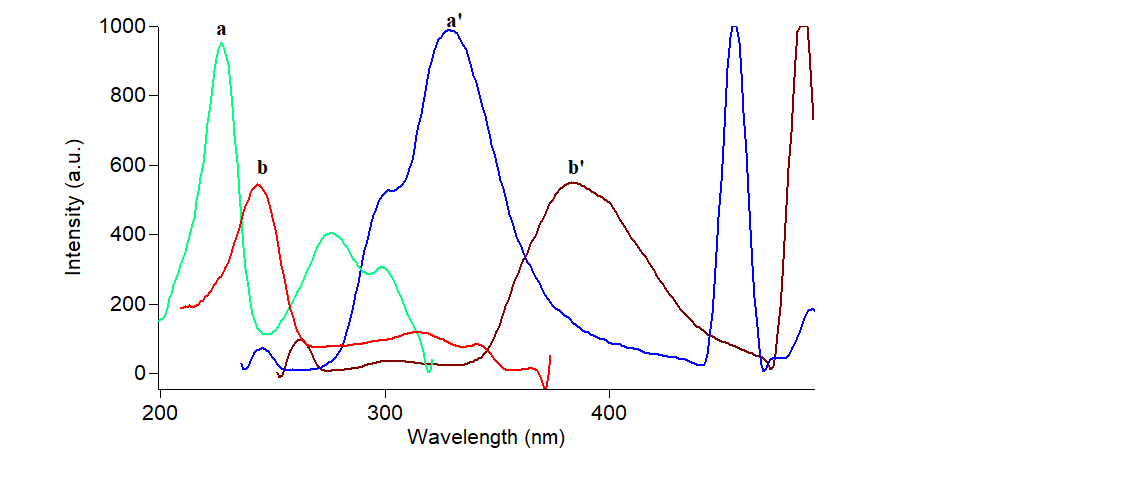


**Figure S1**. Excitation and emission spectra of MLT (20.0 ng·mL⁻¹) (a, a’) and ZOL (10.0 ng·mL⁻¹) (b, b’) in methanol.

**(a)**


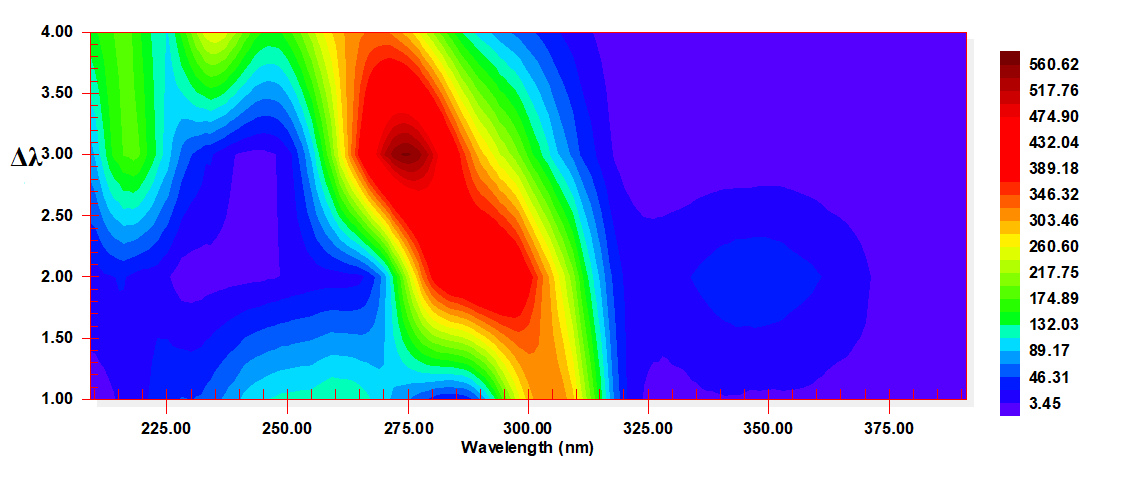

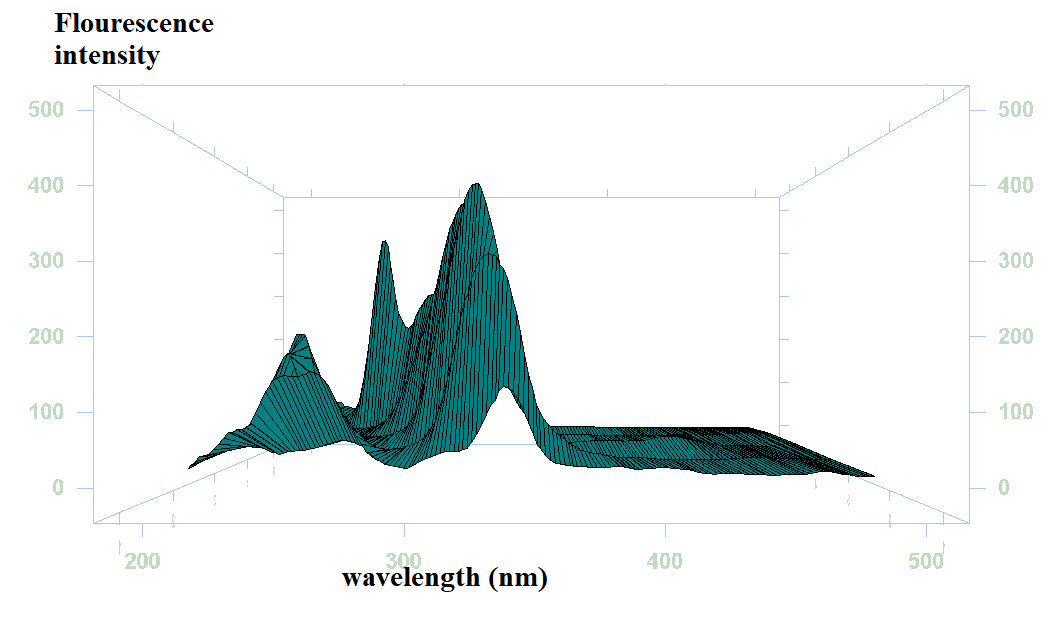


**(b)**


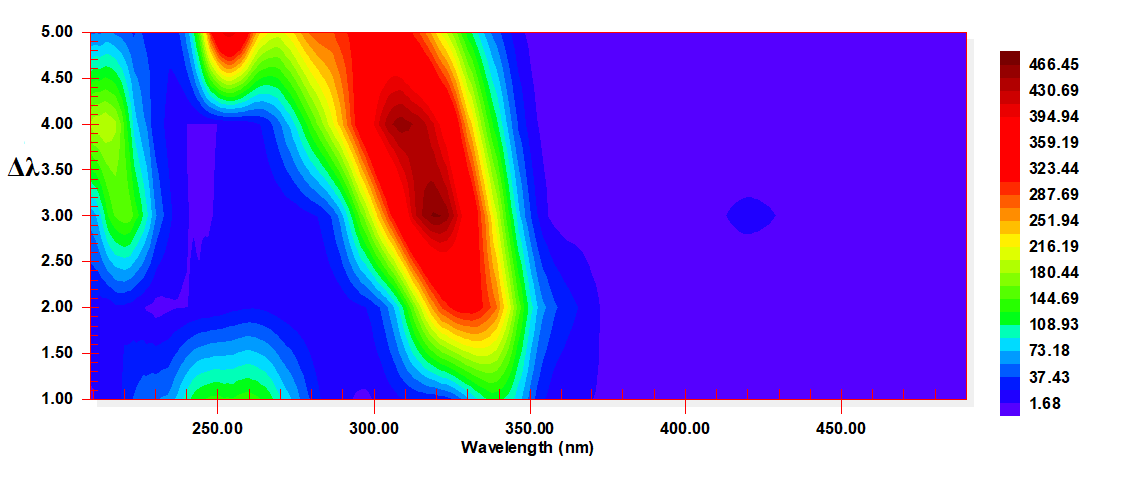

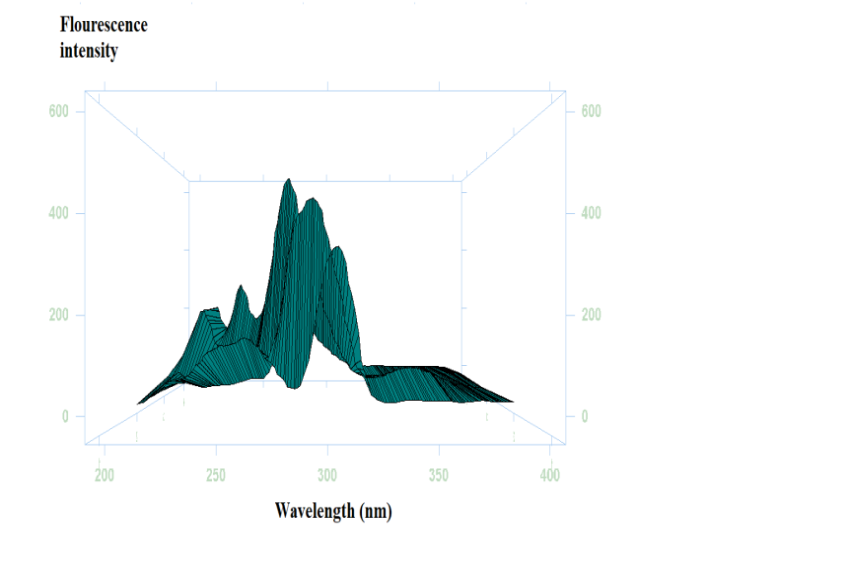


**Figure S2.** Three-dimensional (3D) fluorescence spectra illustrating the effect of different Δλ values on (a) MLT and (b) ZOL.


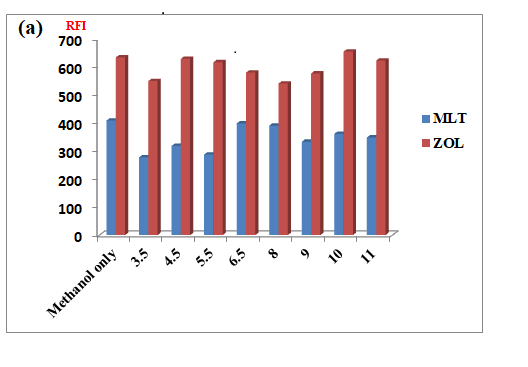

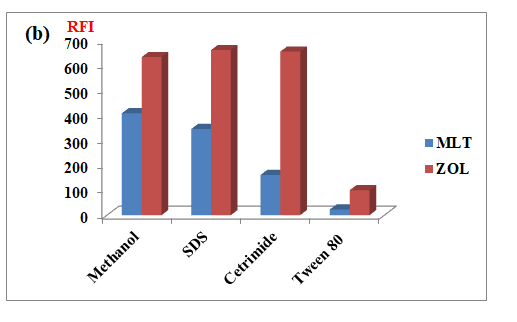

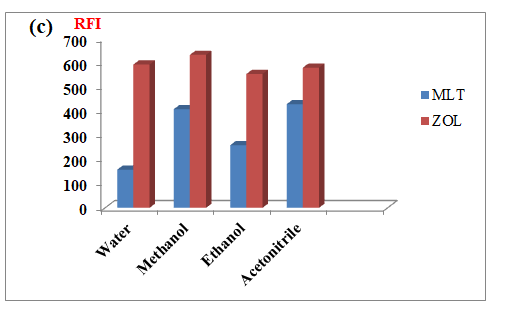


**Figure S3**: Influence of (a) pH, (b) surfactants, and (c) solvents on the relative fluorescence intensity (RFI) of MLT and ZOL (10.0 ng·mL⁻¹ each).


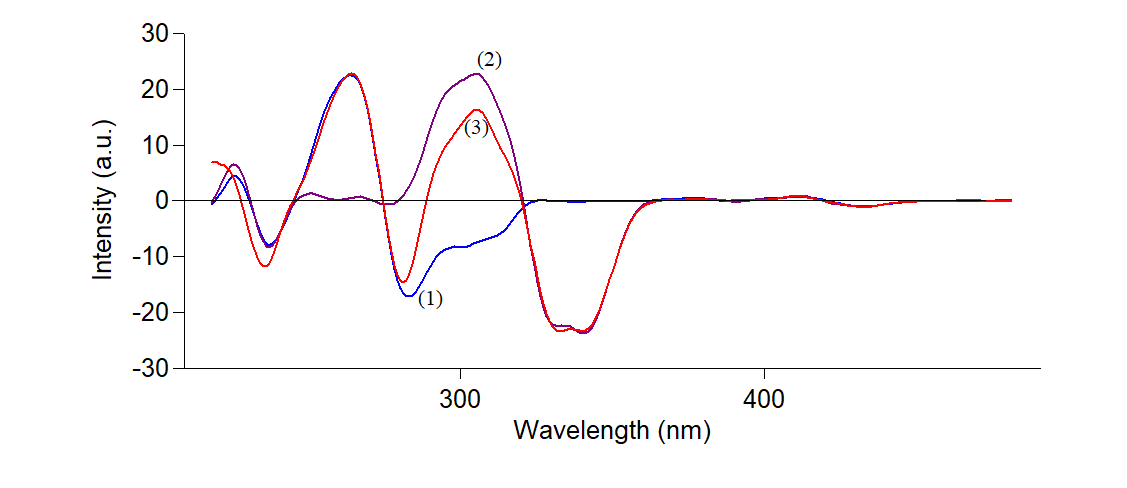
 **Figure S4.** First derivative synchronous fluorescence spectra of: (1) MLT (30.0 ng·mL⁻¹), (2) ZOL (50.0 ng·mL⁻¹), and (3) a mixture of MLT (30.0 ng·mL⁻¹) and ZOL (50.0 ng·mL⁻¹).

**Table S1.** Comparison of the analytical performance of the proposed method with previously reported methods

| **Method** | **MLT** | | | **ZOL** | | | **Ref** |
| --- | --- | --- | --- | --- | --- | --- | --- |
|  | **Linearity range** | **LOD** | **LOQ** | **Linearity range** | **LOD** | **LOQ** |  |
| **HPLC-PDAD** | 6–42 µg/mL | 0.031 µg/mL | 0.108 µg/mL | 10-70  µg/ml | 0.2 µg/mL | 0.6 µg/mL | 1 |
| **HPLC-UV** | 12-72 µg/mL | 0.7729 µg/mL | 2.3422 µg/mL | 20-120 µg/mL | 1.9 µg/mL | 5.9 µg/mL | 2 |
| **Spectrophotometry** | 3-10.5 µg/ml | 0.118 µg/ml | 0.359 µg/ml | 5-17.5 µg/ml | 0.4 µg/ml | 1.3  µg/ml | 3 |
| **Spectrofluorimetry** | 100-1000  ng/mL | 25 ng/mL | 50  ng/mL |  | | | 4 |
| **Proposed FD-SFS** | 8 – 70 ng/mL | 1.6 ng/mL | 4.9  ng/mL | 10.0- 80.0 ng/ mL | 1.2 ng/mL | 3.6  ng/mL | Current work |
| **Proposed HPLC-FD** | 150-1500 ng/mL | 18.9 ng/mL | 57.2 ng/mL. | 50-700 ng/mL | 8.9 ng/mL | 26.9 ng/mL |  |

**Table S2.** Analytical performance characteristics of the proposed methods.

| **HPLC-FD** | | **FD-SFS** | |  |
| --- | --- | --- | --- | --- |
| ZOL | MLT | ZOL | MLT | **Parameter** |
| ex=243, λem=383λ | | Δ λ= 60 nm | | **Wavelength** |
| 50-700 ng/mL | 150.0-1500 ng/mL | 10.0- 80.0 ng/ mL | 8.0- 70.0 ng/mL | **Linearity range (ng/mL)** |
| -0.9301 | -0.031 | 0.1598 | 1.0322 | **Intercept (a)** |
| 0.7218 | 0.0926 | 0.4596 | 0.6769 | **Slope (b)** |
| 0.9999 | 0.9999 | 0.9999 | 0.9997 | **Correlation coefficient (r)** |
| 2.8387 | 0.7201 | 0.2081 | 0.4017 | **S.D. of residuals (S_y/x_)** |
| 1.9387 | 0.5293 | 0.1659 | 0.3318 | **S.D. of intercept (S_a_)** |
| 0.00502 | 0.0006 | 0.0035 | 0.0074 | **S.D. of slope (S_b_)** |
| 8.86 | 18.87 | 1.19 | 1.62 | **Limit of detection, LOD (ng/mL)** |
| 26.86 | 57.17 | 3.61 | 4.9 | **Limit of quantitation, LOQ (ng/mL)** |

**Table S3.** Repeatability and intermediate precision of the proposed methods for MLT and ZOL.

|  | **Concentration (ng/mL)** | **Repeatability** | | | | **Intermediate precision** | | |
| --- | --- | --- | --- | --- | --- | --- | --- | --- |
|  |  | **Mean ± SD** | **% RSD** | | **% Error** | **Mean ± SD** | **% RSD** | **% Error** |
| **FD-SFS method** | | | | | | | | |
| **MLT** | 30.0 | 100.66±1.17 | 1.16 | | 0.67 | 100.63±0.57 | 0.57 | 0.33 |
|  | 50.0 | 101.01±0.57 | 0.56 | | 0.32 | 99.97±1.23 | 1.23 | 0.71 |
|  | 60.0 | 98.41±0.49 | 0.5 | | 0.29 | 100.38±1.61 | 1.61 | 0.93 |
| **ZOL** | 30.0 | 99.75±1.34 | 1.35 | | 0.78 | 99.9±1.51 | 1.51 | 0.87 |
|  | 40.0 | 100.85±1.63 | 1.62 | | 0.94 | 99.94±0.83 | 0.83 | 0.48 |
|  | 50.0 | 99.25±0.66 | 0.67 | | 0.39 | 99.13±0.87 | 0.88 | 0.51 |
| **HPLC-FD method** | | | | | | | | |
| **MLT** | 150.0 | 98.29±0.45 | | 0.46 | 0.27 | 99.28±1.27 | 1.29 | 0.74 |
|  | 300.0 | 102.16±0.48 | | 0.47 | 0.27 | 101.2±0.52 | 0.51 | 0.29 |
|  | 900.0 | 100.97±0.25 | | 0.25 | 0.14 | 99.97±1.29 | 1.29 | 0.74 |
| **ZOL** | 300.0 | 99.6±0.75 | | 0.75 | 0.43 | 99.88±0.43 | 0.43 | 0.25 |
|  | 400.0 | 99.12±1.38 | | 1.39 | 0.8 | 99.28±0.33 | 0.33 | 0.19 |
|  | 500.0 | 98.18±0.42 | | 0.43 | 0.25 | 98.68±0.73 | 0.73 | 0.42 |

**Table S4.** Determination of MLT and ZOL in synthetic mixtures using the proposed methods.

| **HPLC-FD method** | | | | **FD-SFS method** | | | |  |
| --- | --- | --- | --- | --- | --- | --- | --- | --- |
| **% recovery^a^** | | **conc. taken**  **(ng/mL)** | | **% recovery^a^** | | **conc. taken**  **(ng/mL)** | | **Mix no.** |
| ZOL | MLT | ZOL | MLT | ZOL | MLT | ZOL | MLT |  |
| 99.26 | 100.66 | 300.0 | 150.0 | 98.67 | 101.7 | 40.0 | 20.0 | **1** |
| 98.18 | 101.82 | 500.0 | 300.0 | 100.26 | 100.14 | 50.0 | 15.0 | **2** |
| 97.76 | 100.52 | 500.0 | 150.0 | 101.13 | 101.35 | 50.0 | 30.0 | **3** |
| 98.4 | 101.00 |  | | 100.02 | 101.06 |  | | **Mean** |
| 0.77 | 0.71 |  |  | 1.25 | 0.82 |  |  | **± S.D.** |
| 0.78 | 0.71 |  |  | 1.25 | 0.81 |  |  | **%RSD** |

^a^Mean of three determinations.

**Table S5.** System suitability parameters for the proposed HPLC method.

| **Parameter** | **MLT** | **ZOL** |
| --- | --- | --- |
| **Number of theoretical plates** | 1886 | 2748 |
| **Selectivity factor, ɑ** | 1.55 | |
| **Resolution, R_S_** | 5.15 | |
| **Tailing factor, T** | 0.99 | 1.3 |
| **Retention time, t_R_** | 1.78 | 2.84 |

**Table S6.** Application of the proposed method to the determination of MLT and ZOL in their pharmaceutical formulations.

| **ZOL prepared tablets** | | | | | **Dozova melatonin® capsules** | | | | **Parameters** |
| --- | --- | --- | --- | --- | --- | --- | --- | --- | --- |
| **Comparison method [2]** | **HPLC-FD** | | **FD-SFS** | | **Comparison method [1]** | **HPLC** | **FDSFS** | |  |
| 101.65 | 102.01 | | 98.79 | | 102.26 | 98.74 | 100.96 | | **Percentage found^a^** |
| 99.38 | 100.89 | | 100.38 | | 98.89 | 101.53 | 101.84 | |  |
| 100.45 | 99.09 | | 100.2 | | 99.35 | 99.5 | 100.87 | |  |
| 100.49±1.14 | 100.66±1.47 | | 99.79±0.87 | | 100.17±1.83 | 99.92±1.45 | 101.22±0.54 | | **Mean ± S.D.** |
|  | | 0.16 (2.78)* | | 0.85 (2.78)* |  | 0.19 (2.78)* | | 0.95 (2.78)* | **t^b^ test** |
|  |  | 1.66 (19.00)* | | 1.72 (19.00)* |  | 1.59 (19.00)* | | 11.48 (19.00)* | **F^b^ test** |

^a^ Average of 3 replicate determinations.

^b^ The values between parentheses are the tabulated values of *t* and *F* at *P* = 0.05 [5].

**Table S7.** Determination of MLT and ZOL in the prepared co-formulated tablet.

| **HPLC-FD method** | | | | **FD-SFS method** | | | | **Parameters** |
| --- | --- | --- | --- | --- | --- | --- | --- | --- |
| **ZOL** | | **MLT** | | **ZOL** | | **MLT** | |
| % recovery | Amount taken (ng/mL) | % recovery | Amount taken (ng/mL) | % recovery | Amount taken  (ng/mL) | % recovery | Amount taken (ng/mL) |
| 99.46 | 500.0 | 99.84 | 300.0 | 98.2 | 30.0 | 100 | 18 | **Prepared co-formulated tablet (ratio 3:5)** |
| 99.38 | 600.0 | 98.78 | 360.0 | 100.85 | 40.0 | 102.4 | 24 |
| 98.82 | 700.0 | 97.85 | 420.0 | 98.52 | 50.0 | 101.25 | 30 |
| 99.22±0.35 |  | 98.82±1.0 |  | 99.19±1.45 |  | 101.22±1.2 |  | **Mean ± S.D.** |
| 98.53 | 500.0 | 97.31 | 150.0 | 101.39 | 40.0 | 99.32 | 12 | **Prepared co-formulated tablet (ratio 3:10)** |
| 98.34 | 600.0 | 100.01 | 180.0 | 98.42 | 60.0 | 101.51 | 18 |
| 99.35 | 700.0 | 98.34 | 210.0 | 99.38 | 80.0 | 98.91 | 24 |
| 98.74±0.54 |  | 98.55±1.36 |  | 99.73±1.52 |  | 99.91±1.4 |  | **Mean ± S.D.** |

**Table S8.** Analytical eco-scale of the proposed methods

| **Methods** | | **Proposed FD-SFS** | **Proposed HPLC-FD** |
| --- | --- | --- | --- |
| **Reagents** | **Methanol** | 12 | 6 |
|  | **Triethylamine** | - | 6 |
| **Instrument** | **Energy** | 0 | 1 |
|  | **Occupational hazard** | 0 | 0 |
|  | **Waste** | 6 | 6 |
| **Total penalty point**  **Eco-scale score** | | 18 | 19 |
|  |  | 82 | 81 |

**References:**

[1] G. Akula, Y. Talari, S. Phanindra, G. Akula, Method development and Validation for simultaneous estimation of Melatonin and Zolpidem tartrate by using RP-HPLC, Sch Acad J Pharm, 4 (2015) 240-244.

[2] A. Sattar, A. Suneetha, A validated RP-HPLC method for the determination of melatonin and zolpidem tartarate in bulk and pharmaceutical dosage forms, Int Res J Pharm, 9 (2018) 90-96.

[3] T. Venkatachalam, K. Lalitha, Spectrophotometric Methods For Simultaneous Estimation Of Melatonin And Zolpidem From The Combined Tablet Dosage Form, Pharmacophore, 5 (2014).

[4] V. Pucci, A. Ferranti, R. Mandrioli, M.A. Raggi, Determination of melatonin in commercial preparations by micellar electrokinetic chromatography and spectrofluorimetry, Analytica chimica acta, 488 (2003) 97-105.

[5] J. Miller, J.C. Miller, Statistics and chemometrics for analytical chemistry, Pearson education, 2018.
